# New Insights into the Diagnosis and Age Determination of Retinal Hemorrhages from Abusive Head Trauma: A Systematic Review

**DOI:** 10.3390/diagnostics13101722

**Published:** 2023-05-12

**Authors:** Nicola Di Fazio, Giuseppe Delogu, Donato Morena, Luigi Cipolloni, Matteo Scopetti, Sara Mazzilli, Paola Frati, Vittorio Fineschi

**Affiliations:** 1 Department of Anatomical, Histological, Forensic and Orthopaedic Science, Sapienza University of Rome, 00185 Rome, Italy; nicola.difazio@uniroma1.it (N.D.F.); giuseppe.delogu@uniroma1.it (G.D.); donato.morena@uniroma1.it (D.M.); sara.mazzilli@uniroma1.it (S.M.); paola.frati@uniroma1.it (P.F.); 2 Department of Clinical and Experimental Medicine, Section of Legal Medicine, University of Foggia, 71100 Foggia, Italy; luigi.cipolloni@unifg.it; 3 Department of Medical Surgical Sciences and Translational Medicine, Sapienza University of Rome, 00189 Rome, Italy; matteo.scopetti@uniroma1.it

**Keywords:** abusive head trauma, child abuse, retinal hemorrhage, optic nerve hemorrhage, shaken baby syndrome, forensic pathology, ophthalmoscopy, autopsy, immunohistochemistry, operative framework

## Abstract

(1) Background: Head trauma represents the first cause of death in abused children, but diagnostic knowledge is still limited. The characteristic findings of abusive head trauma (AHT) are retinal hemorrhages (RH) and additional ocular findings, including optic nerve hemorrhages (ONH). However, etiological diagnosis must be cautious. (2) Methods: The Preferred Reporting Items for Systematic Review (PRISMA) standards were employed, and the research focus was the current gold standard in the diagnosis and timing of abusive RH. (3) Results: Sixteen articles were included for qualitative synthesis. The importance of an early instrumental ophthalmological assessment emerged in subjects with a high suspicion of AHT, with attention to the localization, laterality, and morphology of the findings. Sometimes it is possible to observe the fundus even in deceased subjects, but the current techniques of choice consist of Magnetic Resonance Imaging and Computed Tomography, also useful for the timing of the lesion, the autopsy, and the histological investigation, especially if performed with the use of immunohistochemical reactants against erythrocytes, leukocytes, and ischemic nerve cells. (4) Conclusions: The present review has made it possible to build an operational framework for the diagnosis and timing of cases of abusive retinal damage, but further research in the field is needed.

## 1. Introduction

Retinal hemorrhage is a pathological finding attributable to numerous conditions, including child abuse. Anatomical structures at a pediatric age, in fact, have different characteristics from those of adults and, therefore, are particularly exposed to damage from the application of external forces, including in cases of child abuse [1].

Child abuse conducted through physical violence has been an entity described in forensic medicine since the 19th century, when Ambroise Tardieu recognized a recurring pattern among infant victims of violence, including meningeal hemorrhages and parenchymal brain damage [2]. Over the decades, numerous clinical, anatomopathological, and radiographic studies have been carried out to identify the main mechanism of damage production; in 1972, following the observation of neurocranial lesion patterns in the absence of external head injuries, Caffey et al. first hypothesized a kinetic mechanism of shaking and coined the term Whiplash Shaken Infant Syndrome [3]. In other words, the damage caused by the application of forces of acceleration, deceleration, and rotation to the atlanto-occipital joint of the child acquired its own dignity in the forensic field and, following further biochemical and clinical investigations, Duhaime et al. introduced the term Shaken Baby Syndrome (SBS) in 1987 [4].

Subsequently, a fundamental role was also recognized of the impact mechanism in determining the observed lesions; the concept of SBS has therefore entered medical practice as a well-defined entity [5,6,7,8,9,10,11,12].

Recently, a new terminology has been proposed by the American Academy of Pediatrics (AAP), namely Abusive Head Trauma (AHT) [13]. This entity encompasses a considerable number of different mechanisms, including “traumatic shaking”. More specifically, AHT is defined as a head or intracranial injury in a newborn or child (<5 years of age) secondary to a blunt impact and/or violent shaking [14].

### 1.1. Epidemiology and Social Burden

From an epidemiological perspective, cranial trauma is the leading cause of death in individuals under 12 months of age [15], and in cases of child abuse, it is the leading cause of death regardless of age [16]. The incidence of this phenomenon worldwide is particularly difficult to estimate, as it is influenced by the parents’ reluctance to seek medical attention and the difficulty of attributing the observed injuries to abusive situations [17,18,19].

It is estimated that 28 out of 100,000 newborns are victims of AHT (Abusive Head Trauma) during their first year of life, with a mortality rate of 21.4%, and half of the survivors have residual disabilities [10]. Therefore, in the USA, there are about 500 annual deaths due to AHT, although this data may be underestimated due to the nature of the underlying crime, which often results in non-reporting by the perpetrators.

Abusive individuals are generally male, identified in the majority of cases as the victim’s father or mother’s boyfriend (37% and 20.5%, respectively), followed by female babysitters (17.3%) and mothers (12.6%) [20,21,22].

Factors that increase the risk of neonatal AHT include male sex, prematurity, low birth weight, disability, age below 12 months, having more than two siblings, maternal smoking during pregnancy, young parents, unmarried parents, and low educational level. All these factors, except for gender, are associated with a lower socio-economic status. However, the absence of these factors does not rule out the possibility of AHT [23].

### 1.2. Referral Difficulties and Valuative Frameworks

Despite the in-depth knowledge of this socio-medical phenomenon, it is estimated that about one-third of AHT cases are not initially diagnosed, especially due to the lack of external evidence of injuries [24]. Moreover, depending on the severity of the injury and the child’s developmental age, these symptoms can be evident and pronounced or subtle and nonspecific. In fact, newborns and young children often have nonspecific symptoms, such as vomiting, which can be misinterpreted or even overlooked by clinicians [25]. An element highly suggestive of maltreatment is a circumstantial reconstruction of events, generally provided by the parent or caregiver, which is incomplete or even in contrast with clinically observed findings (such as a history of a brief fall that caused a clinically significant intracranial injury) [26].

For this reason, numerous frameworks have been proposed to facilitate diagnosis; the pathological triad consisting of subdural hematoma (SDH), encephalopathy, and retinal hemorrhage (RH) probably represents the most classical scheme [27,28]. A clinical screening tool proposed by the Pediatric Brain Injury Research Network, the PediBIRN-4, has been studied using four clinical variables, and is to be used in high-risk patients in intensive care units (ICUs):

- any clinically significant respiratory compromise at the scene of injury, during transport, in the emergency department (ED), or prior to admission;
- any bruising involving the child’s torso, ear(s), or neck;
- any subdural hemorrhages or fluid collections that are bilateral or interhemispheric;
- any skull fracture(s) other than an isolated, unilateral, nondiastatic, linear, parietal skull fracture that shows high values of sensitivity and specificity [29].

Another model was recently proposed by Iyer and Lemos and was entirely based on ophthalmoscopic assessments of retinal hemorrhages in suspected AHT patients [30]. 

Obviously, such tools are exclusively used clinically based on targeted and obtainable in vivo data and are therefore aimed at detecting cases of child abuse for the safeguarding of minors. In a forensic pathological context, therefore, the diagnostic triad still constitutes an unsurpassed tool.

### 1.3. Triad of Clinical Presentation

Despite the considerable variability in the clinical presentation of individuals who have suffered from child abuse, the statistical prevalence of certain signs has led to the identification of a clinical triad [27,28]:

- SDH;
- Encephalopathy;
- RH.

Understanding the genesis of such injuries, as already mentioned, requires a basic knowledge of the anatomical differences between the neonatal and adult brain; firstly, the neonatal brain has a higher percentage of water composition, a lower degree of myelination, and a more pronounced subarachnoid space [31]. Therefore, due to the greater specific weight and asynchronous movement between the skull and brain, the latter is more exposed to concussions and counterblow mechanism traumas, as well as more vulnerable to violent shaking. The rupture of bridging veins, as in adults, and the rupture of capillaries seem to be the main pathogenetic mechanism of SDH [32]. This observation, highly frequent in cases of abuse, finds a suggestive topographic distribution affecting the fornix, interhemispheric fissure, and cerebellar tentorium [33]. Of course, depending on whether the trauma is due to a direct impact on the infant or a shaking mechanism, such findings may or may not be accompanied by external injuries affecting the skin, muscular, and skeletal tissues [34]. In addition, in cases characterized by chronic abuse, previous SDH may show outcomes consisting of neocavities filled with cerebrospinal fluid (CSF) called subdural hygromas [35].

Regardless of the type of trauma, stretching of the medulla oblongata, neuropraxia of the fibers contained within the white matter, and cerebral edema appear to be the main culprits for the genesis of encephalopathy, which clinically manifests as lethargy, seizures, and dyspnea. In addition, the increase in intracranial pressure (ICP) can precipitate the clinical picture with diffuse ischemic damage and compromise the bulbar centers responsible for breathing [34].

Retinal hemorrhagic patterns, on the other hand, constitute a numerous entity and are characterized by a considerable number of possible pathogenetic mechanisms.

Retinal Hemorrhages

Retinal hemorrhages are a core manifestation in abused children, being present in about 85% of cases [36,37]. The eye, due to its peculiar anatomical condition, is indeed the target of both direct and indirect physical insults.

Especially in children younger than 6 months, a non-accidental traumatic etiology is strictly correlated to severe retinal hemorrhages. According to the literature, pediatric retinal hemorrhages may be triggered by several mechanisms. Trauma represents the most frequent cause of retinal hemorrhages, especially birth trauma, but in children older than a few weeks, AHT still represents the main cause [38,39]. Among other causes, an intra-cranial pressure increase, systemic and local pathologies (e.g., coagulopathy and leukemia), and cardio-pulmonary resuscitation can also be found [40].

Due to the paucity of AHT-related symptoms, considering the usual incompleteness of anamnestic information, about 30% of abused children are misdiagnosed at first access to medical care. Additionally, the literature shows that 25% of children are going to suffer further abuse before being correctly diagnosed with AHT [41]. Therefore, whenever the collected medical history is unclear and reveals to be incongruous with the child’s injuries, an AHT should always be suspected [42].

Scientific evidence suggests that the association of retinoschisis and retinal folds whenever there is no history of violent accidental trauma has a 100% positive predictive value for AHT [36,43].

Several pathogenetic mechanisms of retinal hemorrhages have been described:

- The most ancient theory, undoubtedly, is the mechanism proposed as “Terson’s Syndrome” [44,45], according to which an increase in ICP causes a hydraulic transmission of the CSF pressure to the eye through the laminae of the optic nerve. In this way, an increase in intraocular pressure (IOP) above the intravascular pressure of the capillary and venous vessels of the ocular coats produce a reduction in blood flow or a complete interruption of circulation; the resulting ischemic insult, affecting both the eye, the dura mater, and the brain, produces a rupture of the thin monolayered endothelial layer of the capillaries and, therefore, a hemorrhage [46]. Additionally, the central optical vein is directly affected by the increased pressure inside the optic nerve, primarily causing congestion of the entire retinal vascular bed [47]. The extent and degree of the leak also directly depends on the extent of the pressure and ischemic insult [48]. However, the low incidence of papilledema in relation to the RH finding would seem to discredit this theory [28];
- Along with this theory, an additional hydraulic mechanism has been proposed, whereby the venous and capillary pressures of the retinal vessels undergo a sudden increase due to the transmission of force from the vitreous to the retina during shaking, causing its rupture [49];
- The theory of vitreoretinal traction is currently the most well known [50], supported by the fact that most hemorrhages are located where retina and vitreous are deeply attached. In theory, due to the intense production of rotational and linear movements with sudden acceleration and deceleration, the vitreous exerts a shearing force on the retina, causing a mechanical rupture of the vessels. Although according to numerous authors, this theory is undermined by the lack of supporting evidence [51]; the scientific plausibility of the proposed mechanism is reflected in the frequent finding of another highly suggestive ocular findings in AHT, i.e., retinoschisis and macular folds [52]. Circular ruptures of the retinal epithelium are in fact highly sensitive findings for AHT, but not specific; they can also be found in cases of direct ocular trauma or SDH. From a morphological point of view, this finding can assume varied aspects: it can in fact present itself as continuous or discontinuous; partial or complete; and peri-macular, trans-macular, or peripheral and often it appears surrounded by a hyperpigmented or frankly hemorrhagic linear ring [43]. In addition to such lesions, it is possible to observe dispersion of blood inside the vitreous (vitreous hemorrhage), a critical element as it often masks underlying lesions under ophthalmoscopic observations.

Thus, in order to formulate a differential diagnosis between accidental head trauma and AHT, some specific features of retinal hemorrhages should be evaluated, such as retinal region, subtype, and number of hemorrhages, as well as retinoschisis and retinal folds. In AHT cases, different types of hemorrhages have been described; in less severe cases, they occurred within the posterior retinal region and were single layered, while in more severe cases, hemorrhages were multi-layered and they spread up to the ora serrata.

In conclusion, there are three categories of retinal hemorrhages: preretinal (associated with vitreous hemorrhage), intraretinal, and subretinal. Thus, hemorrhage severity is directly related to the severity of the trauma [53,54].

From a temporal point of view, a significant gap has been observed between the resolution times of intraretinal hemorrhages, which in most cases require only a few days to disappear completely, and those of preretinal and subretinal hemorrhages, which can take weeks or even months to resolve, depending on the extent and severity of the lesion. This difference in resolution time can be useful for the diagnosis of chronicity when the hemorrhages are visible in different stages of resolution [43].

Although AHT hemorrhages are generally bilateral, an eventual finding of a unilateral hemorrhage does not exclude any diagnosis of traumatic injury; furthermore, studies have not found a significant correlation between the laterality of intracranial and retinal injuries [28].

If the ophthalmoscopic evaluation reports many severe, multi-layered hemorrhages, which have spread to peripheral areas of the retina whose etiology cannot be verified, an AHT diagnosis should be made unless proven otherwise. In mild cases, instead, AHT should always be considered in the differential diagnosis [55] (Table 1).

Guidelines from the American Academy of Pediatrics state the necessity to perform an indirect ocular examination, led by a skilled ophthalmologist, to evaluate the presence of retinal hemorrhages in children or infants whose abuse is suspected [56].

Despite the fact that the forensic practice has consolidated the use of the triad for the detection of AHT, many recent studies have criticized its actual value. In particular, numerous sources attribute an apparent statistical significance of such signs to biases of circular reasoning [57,58]. Moreover, the neonatal age would be more prone to the development of SDH compared to later stages of infancy [59]; therefore, the incidence of such a condition would be linked to a selection bias. In addition, even in 1987, Duhaime et al. [4] argued that shaking alone was insufficient to determine retinal lesions; therefore, numerous animal [60,61,62] and mannequin experiments [63,64] have sought to find an answer to this question. A review of the international scientific literature in 2017, analyzing a total of 3773 articles, illustrated the limited scientific evidence currently available due to the strong bias found [65]; however, further studies have also found critical issues in the methodology of the review itself, affecting its conclusions [66]. Therefore, the current scientific orientation tends to attribute considerable diagnostic weight to RH in the context of an AHT diagnosis, provided that state-of-the-art investigations are carried out. Furthermore, based on careful evaluations of available cases, the diagnostic triad for AHT has regained probative validity [57].

### 1.4. Other Ocular Findings Compatible with AHT

RH is not the only finding associated with AHT; in cases of direct eye trauma, for example, it is possible to observe subconjunctival lacerations or hemorrhages. However, it is important to remember that this finding can also be secondary to an increase in intrathoracic pressure which, in addition to maltreatment, can also be due to a Valsalva maneuver or prolonged vomiting. It is also important to exclude the possibility of predisposing clinical conditions such as coagulopathies. Furthermore, in cases of direct trauma to the eyeball, consequences can include eye rupture and direct damage to the cornea, such as abrasions and lacerations observable as opacities upon direct observation.

Another consequence can be hyphema, or bleeding in the anterior chamber of the eye, and iatrogenic cataracts from dislocation of the lens due to rupture of the suspensory apparatus. Due to the numerous conditions associated with AHT affecting the anterior segment of the eye, an in vivo or post-mortem evaluation without delay (due to the consecutive corneal opacification phenomena) should be performed using a slit-lamp biomicroscope [43] or post-mortem monocular indirect ophthalmoscopy (PMIO) [67], which, however, is currently poorly available due to the high costs.

The posterior section of the eye can also be particularly useful for detecting lesions associated with maltreatment; in suspicious cases, therefore, enucleation of the eye in an autopsy setting is recommended for the observation of posterior structures otherwise not visible [28,68]. Among the phenomena typically associated with AHT affecting the posterior district, it is possible to include optic nerve sheath hemorrhages (ONSH) [69], whose genesis is still debated. Such hemorrhages typically involve the anterior portion of the optic nerve, involving multiple layers but, more frequently, the subdural layer [70] and, in decreasing order of frequency, the epidural, intradural, and subarachnoid layers [69]. The genesis of these hemorrhagic phenomena, according to some authors, can be attributed to the translational–rotational accelerations to which the nerve is subjected to during shaking [71] and the fragility of the bridging veins afferent to the retinal central vein; therefore, an abnormal linear or rotational acceleration of traumatic origin can currently be considered the most accredited etiopathogenic mechanism [72].

On the other hand, an origin of a hydrodynamic/pressure nature similar to that proposed for RH is not supported by evidence of a linear compromise of the Glasgow Coma Scale at patient discharge and the degree of ONSH, nor by the finding of a correlation between increased ICP and severity of the condition [28,73]. In any case, according to some authors, ONSH can be characterized by a stronger correlation with AHT than with RH (odds ratio = 32.5 vs. odds ratio = 18.9; *p* = 0.001) [74]; however, this strong correlation appears to be attributable exclusively to cases of non-contact injury, as the rotational acceleration typical of shaking would be more effective in the genesis of this phenomenon. Furthermore, in cases of high-energy traumas, the frequent coexistence of diffuse axonal injuries (DAI), SDHs, or skull fractures allow for an easy differential diagnosis [75].

Finally, rapid accelerations and decelerations can also damage the accessory structures of the eyeball, producing typical aspects such as periorbital fat or extrinsic muscle hemorrhages [76]. In order to highlight retro-ocular hemorrhagic phenomena in living subjects, it is useful to consider the undoubted usefulness of an MRI investigation conducted in gradient echo T2 (GRE T2-w) or susceptibility-weighted imaging (SWI) sequences, which have a high diagnostic sensitivity and specificity but are not very useful for dating phenomena [77,78].

### 1.5. Differential Diagnosis of RH

As already mentioned, numerous conditions can cause damage to the retina and other ocular structures (Table 2).

Therefore, RH must be carefully examined, documented, and correlated with the patient’s medical history; it is a diagnosis of exclusion that can only be achieved through the application of rigorous scientific methods that, in the context of a judicial process, can lead to correct conclusions with high levels of specificity [43].

Given the aspects shown so far, it is reasonable to ask whether a traditional clinical and forensic pathological assessment of RH is sufficient to diagnose AHT within a sufficiently high probability margin to constitute valid evidence in a judicial context. The pathological triad described, in fact, has many weaknesses and excludes many tools that a multidisciplinary approach can provide.

The purpose of this review, therefore, is to analyze the most recent aspects proposed by the scientific literature in order to construct an action plan for the pathologist facing cases of particular complexity and sensitivity, providing useful elements for diagnosis and lesion timing.

## 2. Materials and Methods

The present systematic review was prepared according to the Preferred Reporting Items for Systematic Review (PRISMA) standards [85]. The focused question of the present study is: “What is the current gold standard in the diagnosis and timing of retinal hemorrhages in cases of fatal abusive head trauma?”.

### 2.1. Inclusion Criteria

We selected the studies to be included in the present systematic review based on the following characteristics, according to the Population Intervention Comparison Outcome (PICO)model [86]:

- Patients: infant victims of AHT presenting RH independent of fatality of outcome;
- Intervention: clinical and radiological evaluation and/or judicial autopsy completed by histopathological investigations;
- Comparison: evidence prior to the investigated period;
- Outcome: accuracy of methods and indicators used, correspondence with histopathological results, concordance of different studies on RH dating, and enforceability in forensic practice.

### 2.2. Exclusion Criteria

We rejected studies with these characteristics:

- Date of publication prior to 2016;
- Field of interest unrelated to a forensic setting;
- Insufficient innovation in results;
- Unavailability of abstract in the English language;
- Any kind of publication other than articles published in impacted journals.

### 2.3. Information Source and Search Process

A systematic literature search and a critical appraisal of the collected studies were conducted by D.M. and G.D. A bibliographic search using three databases (PubMed, ScienceDirect, and Web of Science) was carried out from 1 January 2016 to 15 August 2022. The articles of interest were analyzed in the full-text version and selected by an initial reviewer (G.D.) and the results were compared with those of a second reviewer and the term (N.D.F), following an open discussion among all authors (N.D.F., G.D., P.F., and V.F.). Articles that respected the inclusion criteria were admitted. Only papers or abstracts in English were included in the search. No unpublished or gray literature was searched.

The search for relevant articles on electronic databases was carried out using the following search criteria: [“Abusive Head Trauma” OR “Abusive Trauma” OR “Shaken Baby Syndrome”] AND “Retinal Hemorrhage” AND “Forensic” in the title, abstract and keywords. The outcome chosen for this systematic review is the “AHT-related retinal hemorrhages diagnosis and age estimation” as well as the validation of the immunohistochemical techniques currently available for this purpose.

## 3. Results

### 3.1. Study Selection

**Figure 1:**
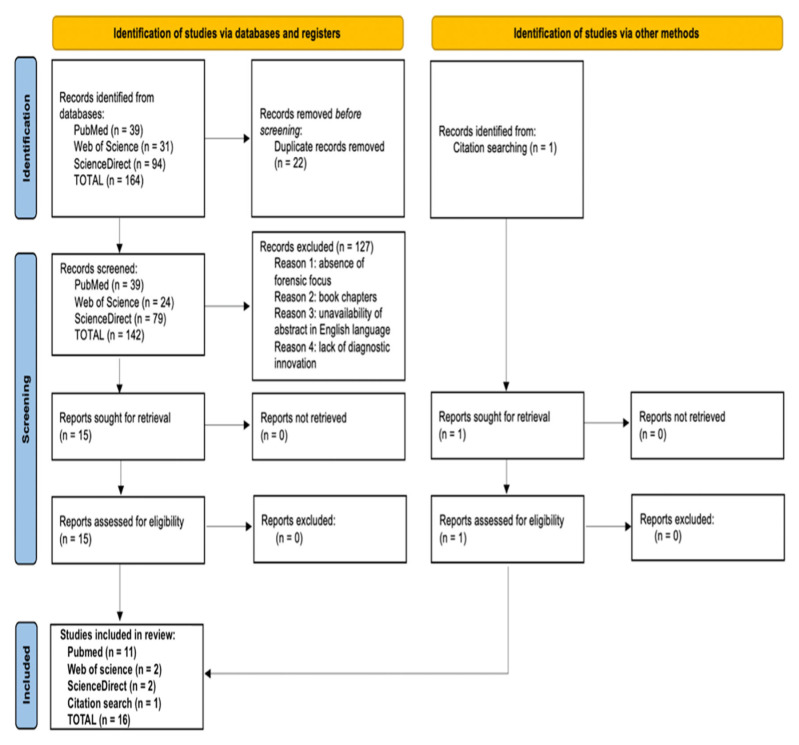
PRISMA 2020 flow diagram [85] applied to the present research.

The database-based search produced a total of 164 articles, of which 22 were duplicates. After excluding duplicate items from the selection, 142 articles were reviewed through their titles and abstracts. Subsequently, 123 articles were excluded because they did not meet the inclusion criteria. Finally, 16 articles were included for qualitative analysis [71,78,87,88,89,90,91,92,93,94,95,96,97,98,99,100] (Figure 1).

### 3.2. Study Characteristics

The 16 included studies were performed in eight countries (Canada, The U.S.A., The Netherlands, France, Germany, Italy, Japan, and Thailand) across three continents between 2016 and 2021. Despite two articles [87,88] being written in German, all of the considered papers presented abstracts written in English. From a structural point of view, six of them consist of case reports or case series associated with a brief literature review [71,87,88,91,92,93]; two articles consist of reviews of the scientific knowledge available at the time of writing [78,98]; one is a technical note of the author [90]; and the seven remaining are original articles, all statistically validated [89,94,95,96,97,99,100].

Except for the review articles, the remaining works account for a complete cohort of 296 infant victims of AHT within the age range from 10 days (0 months) to 1041 days (34.7 months). Different methodologies were pursued among the considered works; clinical examination of alive subjects was executed by means of ophthalmoscopy or a combination of RETcam and OCT [87,88,90,92,97,99]; imaging techniques were employed by means of Magnetic Resonance Imaging (MRI) with different time-weights (3D-SWI, coronal T2-w, T1-w, and axial GRE T2-w protocols) [78,96]; forensic autopsies permitted the direct observation of macroscopic pathologic alterations of the eye and periorbital soft tissues [71,88,98]; and histopathological investigations were based on traditional H&E staining [71,88,91,94,95,98,100], histochemical iron-specific and PAS stains [91,94,95,100], and immunohistochemical reactants (β-APP, ubiquitin, GFAP, Glycophorin A, Nestin, CD44, and collagen type IX) [89,98,100]. The main characteristics of the selected articles are summarized in Table 3.

### 3.3. Risk of Bias

The significance of this review lies in the choice of an area and the diagnosis of AHT based on ocular findings of a hemorrhagic nature, on which substantial uncertainty still weighs due to the limited sensitivity and specificity of the methods used routinely. For this reason, rigorous research of current scientific evidence has allowed us to group together studies that are remarkably heterogeneous and worthy of an overview. It has already been highlighted that of the 16 studies included in the review, only 8 belong to the category of experimental studies, which are further subdivided into retrospective, prospective, monocentric, or multicentric and were all validated by a careful statistical analysis (Table 4). However, case reports or case series containing elements of an innovative nature were not excluded (Table 5) and, for the same reason, a technical note and two reviews (Table 6) were also admitted to the review, one of which was systematically conducted according to PRISMA criteria [85]. Furthermore, a considerable heterogeneity emanates from the geographic regions of origin of the studies, coming from eight countries spread over three continents; however, in consideration of the validity of the protocols used in all countries and based on the knowledge currently available, it is believed that no bias can derive from the race of the individuals analyzed. Between the studies, there is a relatively short time interval, equal to about 6 years (2016–2021), which however involves the need for a critical reading of the results. The same considerations can be extended to the different sizes of the samples taken into consideration, sometimes consisting of a single individual and sometimes consisting of up to 133 infants. No further stratifications based on sex have been performed as no gender differences have yet emerged in the presentation of the disease.

## 4. Discussion

As already extensively stated, the clinical and forensic issues inherent in finding retinal hemorrhages are numerous and long-standing; of these, the immediate classification of a hemorrhagic phenomenon affecting the retinal plane as abuse or violent is of particular importance due to the subsequent initiation of investigations. The observation of the ocular fundus is therefore a vital moment in every single case, and a thorough knowledge of the main findings is necessary. Since the introduction of fundus observation techniques, the purpose of such examinations has been focused on the observation of living subjects. Due to the frequent transience of hemorrhagic findings affecting infant retinas, the American Academy of Pediatrics Council on Child Abuse and Neglect recommends ophthalmological consultation “preferably” within 24 h and “ideally” within 72 h of the reported event (or alleged abuse) [104].

Furthermore, numerous predictive and operational models have been proposed, including that of Iyer and Lemos [30] (Table 1), proposing elements that require ophthalmologic consultation and substantially aiming at promoting greater importance of the bilaterality of RH. Binenbaum and Forbes’ severity scale [52] (Table 2) also seems to focus on this aspect, while not excluding the possibility of homolateral findings.

Regarding the operational methodology used on the living subject, the most widely used method is the observation of the fundus through ophthalmoscopy. Moskwa et al. [97] analyzed ophthalmologic reports of 133 patients in their institution using pharmacologically induced mydriasis and photography of the posterior segment. From this experience, some insights for further investigation emerged, e.g., the laterality of retinal lesions appeared to be correlated with the age at diagnosis, as subjects over 6 months of age more frequently showed bilateral findings compared to those under 6 months of age; these differences can be attributed mainly to anatomical reasons, and therefore the proposals advanced by the previous authors [30,52] would be partially inaccurate as they do not consider the age of the infant at the time of diagnosis. In addition, the laterality of intracranial lesions was not associated with the laterality of ocular lesions, and above all, the limited diagnostic value of bilateral findings compared to unilateral ones emerged.

Even Barth et al. [87] highlight in their case report the possibility of unilateral retinal hemorrhages in victims of AHT, especially if the traumatic insult is located on a specific side. Notably, both reported cases were under 3 months of age, supporting the hypothesis that an age of >6 months is a predisposing factor for the onset of ocular injuries.

Finally, the team of Moskwa et al. [97], in agreement with Fieß et al. [88], emphasized the vast heterogeneity of ophthalmoscopic reports due to the high observer dependence, the high error rate when applied by non-specialists, and the poor sensitivity in detecting peri-equatorial lesions. Ultimately, when possible, they recommend the combined execution of further examinations, including visual field examinations and Optical Computed Tomography (OCT).

Overall, the methods currently used in clinical practice for the ophthalmological screening of AHT, according to Oliva et al. [99], are:

- Slit lamp, in order to ascertain the existence of damage to the anterior segment of the eye and to examine pupillary reflexes in subjects with acute neurological disorders;
- Indirect ophthalmoscopy, a relatively ancient tool to examine the posterior pole of the eye, with a limited focus on the retina but a very convenient cost-effectiveness to justify its wide application nowadays;
- Digital wide-field fundus photography (DWFFP), a technique capable of investigating the whole retina and to catch retinal images; it is considered to be highly economic with a good reliability (100% sensitivity, 85.7% specificity [102]), especially if combined with fluorescin angiography (FA);
- OCT, which is particularly useful to investigate the retinal posterior pole and optical nerve disc, including the different layers possibly showing hemorrhage. It allows the visualization of the vitreoretinal membrane, supporting a diagnosis based on a direct mechanical trauma mechanism.

Oliva et al. applied these techniques as a diagnostic tool for AHT in a clinical setting in living subjects admitted to an Emergency Department. After conducting a medical history collection, a first clinical evaluation, and, subsequently, a brain CT positive for epidural, subdural, and subarachnoid hemorrhages, a preliminary observation of the eye was carried out using a portable slit lamp. Then, an examination without pupil dilation was performed using a hand-held OCT (Bioptogen Leica), followed by a DWFFP with RetCam 3 (Natus, Pleasanton, CA, USA) under pharmacologically induced mydriasis.

The application of OCT in the presented cases allowed the observation of the depth of the hemorrhages and the layers they belong to, as well as the state of the optic disc; conversely, the RetCam technique allowed the observation and documentation of the superficial appearance of the hemorrhages, their extension to the quadrants and beyond the ora serrata, and finally, the assessment of the possible chronicity of the findings.

This study highlights how RetCam (DWFFP) is the current diagnostic gold standard for retinal imaging, allowing for the observation of both eyes and obtaining a field of view of 130 degrees. Furthermore, the possibility of applying a contrast medium (FA) increases the temporal window for diagnosis in cases of late clinical presentation, as well as enabling the visualization of the peripheral retinal vascular bed and the detection of hypoperfused retinal areas. However, as it is a “contact” system, it is poorly tolerated by awake infant patients and potentially dangerous in case of stimulation of the oculo-cardiac reflex due to the significant pressure applied to the eyeball. It is also difficult to perform in the emergency room and is dependent on the pupil diameter.

According to Yusuf et al. [93], the Optos P200MA Scanning Laser Ophthalmoscope (SLO) represents a valid alternative to the RetCam, as it allows obtaining a 200° field of view with a single shot, without the need for eye contact or sedation. Consequently, this tool reduces the risk of stimulating the ocular-cardiac reflex in subjects already affected by intracranial hypertension; furthermore, confocal optics allow the visualization of lesions detected on the retinal plane, including macular schisis and pre-macular hemorrhages. Among the main limitations of the method, one notes the poor accessibility and comfort, particularly due to the low portability and the need to position the infant in the “flying baby” position.

In comparison, OCT is considered an important complementary technique to DWFFP, as it allows for better definition of retinal anatomy and observations, in particular of the vitreoretinal membrane, in cases of direct mechanical trauma. In general, OCT is easier to use, less invasive, and has a lower risk of complications. Consequently, the limitations of RetCam can be minimized through the combined implementation of such instruments. The data presented so far are entirely related to patients who were alive at the time of diagnostic assessments. However, post-mortem visualization of the retinal fundus is a diagnostic frontier currently under experimentation, as in the case of Lantz et al.’s proposal [90].

Following the proposal in the past to use post-mortem monocular indirect ophthalmoscopy (PMIO) for the projection and photography of the posterior fundus of deceased subjects, the major problem encountered was the high cost of a dedicated fundal camera. For this reason, a proposal was made to use modern smartphones for this purpose, due to the increased power of integrated cameras and the ability to capture high-definition videos and use the built-in flash as an illumination source. Methodologically, the application of an indirect lens in the pericorneal position parallel to the retinal plane and aligned with the smartphone’s camera allows the acquisition of iconographic material at a very low cost, with high definition, portability, availability, and shareability, while maintaining high data security through the implementation of data encryption programs.

The application of such a convenient tool in the clinical sphere could, in the future, constitute an important diagnostic and monitoring instrument for numerous pediatric ophthalmic pathologies, as well as greatly expand patient cohorts in any type of study.

In the field of post-mortem diagnoses of RH, the radiological sphere appears considerably more developed than by direct observation of the retina. Currently, due to the clinical need to visualize cranial, vertebral, costal, or long bone fractures, as well as intra- or extra-cranial hemorrhages, CT examination is the most commonly used tool in cases of AHT. Through this procedure, although not characterized by high sensitivity or specificity, RH can be visualized as pinpoint and/or laminar hyperdense foci within the retinal plane [98].

Instead, according to Cartocci et al. [80], MRI could be a valuable tool through the use of gradient echo T2 sequences (GRE T2-w) and susceptibility weighted imaging (SWI) on the eyeball, allowing for the detection of small areas of signal dropout at the retinal level. Furthermore, since such investigations are mainly aimed at studying the central nervous system (CNS), it is possible to appreciate intracranial hemorrhagic lesions through the same weighting, allowing for a fine and complete investigation. Unfortunately, the application of such an investigation provides little information for the purpose of dating the lesion; however, considering the degradation processes of hemoglobin, the observation of the different magnetic susceptibilities of multiple lesions allows to assign them as having the same polychronic origin. However, further studies are necessary to analyze the behavior of blood collections at the ocular level, due to the different concentrations of oxygen and thromboplastin and the consequent different local-regional hemoglobin metabolism [103].

At the vitreal level, on the other hand, an uneven visualization of the lesion pattern represents a useful tool for monitoring the intracavitary hemorrhagic spread and hypothesizing its topographic origin. However, the ocular plane is not the only area of interest in the field of ophthalmological diagnostics of AHT; the optic nerve, similar to the retina, is an extremely useful area of investigation and, in this regard, Zuccoli [96] was the first to apply MRI with the 3D-SWI technique to sagittal and coronal planes to the orbits of patients recovered at his own institution. In this way, it was possible to observe the presence of ONSH through the detection of ONS thickening/3D-SWI hypointensity and by the observation of mass effect lesions at the level of the CSF space. A quantitative definition of hemorrhagic lesions at the level of the ONS has been proposed in the following way:

- Grade 1—moderate deformation in the absence of an increase in nerve diameter;
- Grade 2—focal deformity and a moderate increase in diameter;
- Grade 3—loss of anatomical demarcation of the ONS with a severe increase in diameter.
- A rough temporal diagnosis has been proposed by distinguishing between recent hemorrhages, which are depicted as very dark areas inside the optic nerve parenchyma, and past lesions (hemosiderin deposits), which are described as scattered dark foci on the surface of the optic nerve.
- At the end of a thorough anamnestic and instrumental evaluation, autopsy remains a fundamental tool for dealing with fatal cases of abusive head trauma (AHT) [88]. Specifically, the search for ophthalmological findings requires the execution of a specific sectorial protocol at the cranial level [71]. By extracting the brain through a classic craniotomy, in fact, the exposure of the eye and its vasculo-nervous hilus requires the removal of the orbital roof by carefully cutting through the bone plane with tools of small dimensions, such as scissors or rongeurs. At this point, the exposed conjunctiva can be removed by cutting from the Tenon’s fascia to the sclera.
- At such a point, the eye and optic nerve (ON) become observable within their anatomical dwelling and should be removed en bloc (as for the intracanalicular portion of the ON) and fixed in formalin. Alternatively, an anterior approach is made possible by incising the conjunctiva and extracting the orbital content, which, however, does not allow for the preservation of posterior structures (e.g., the ON) and their observation in situ [98]. In cases of AHT, in fact, the importance of additional findings such as hemorrhagic infiltration involving periorbital adipose tissue, intrinsic musculature, peripapillary sclera, and especially the ON has already been asserted. Regarding peripapillary scleral hemorrhages (PSH), already known in a forensic context to be related to AHT [104,105], it has long been believed to derive from the typical acceleration/deceleration forces of shaking applied to the sclero-papillary junction with consequent traction of the intrascleral vessels of the Zinn’s arterial ring [106]. However, the experience of Oshima et al. [93] has allowed for a new hypothesis to be put forward based on the assumption that bleeding results from the Zinn–Haller arterial ring directly associated with traction of the ON. Consequently, the same author proposed the finding of PSH as an indicator of acceleration trauma stronger than RH.
- Furthermore, as reported by Puanglumyai’s team [71], the visualization of the ON may be obstructed by a local hemorrhagic infarction, which is why non-traumatic means (e.g., clamps) can be applied below the nerve to expose it more clearly. According to the same author, this method allows for rapid and effective in situ visualization of the hemorrhagic findings, sometimes better than after enucleation in the case of a minimal lesion size, which still requires histopathological confirmation. Ultimately, in confirmation of what has already been stated in the literature, the modest case series proposed by Puanglumyai et al. (11 victims of AHT) allowed for an appreciation of the specificity of the ONSH findings equal to 100%, with a topographic predilection for the retrobulbar portion considered to be more subjected to tension forces.
- The diagnostic culmination of an investigation into suspected AHT is currently constituted by histopathological investigations. Among the methods aimed at detecting ophthalmic hemorrhagic lesions, the classic hematoxylin–eosin (H&E) technique can be counted. As illustrated by Fieß et al. [88], in a discussion of their cases, in fact, their investigation conducted on acute retinal lesions allowed the visualization of extravascular red blood cells, distinguishing the different retinal layers affected by the hemorrhages as well as highlighting any folds or detachments (Figure 1).

**Figure 2:**
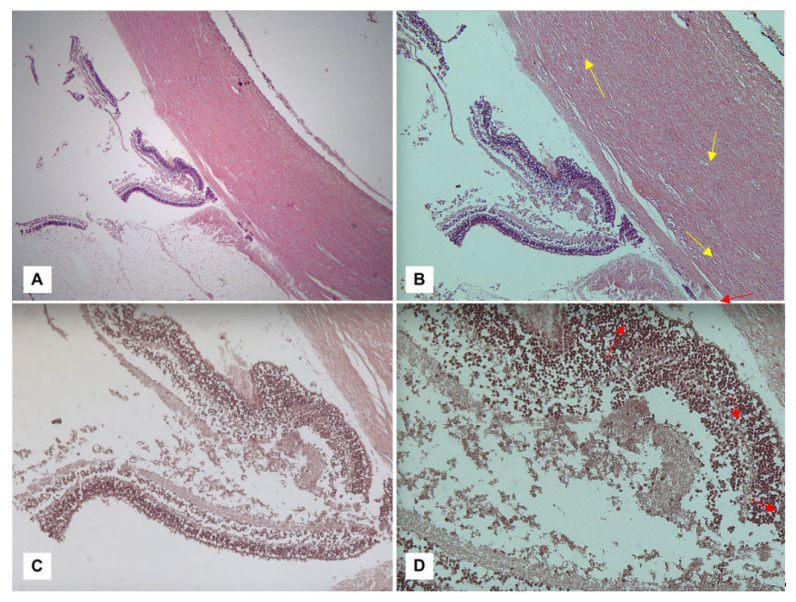
Visualization of retinal tissue stained with H&E at different magnifications: (**A**) 2.5*×*; (**B**) 5*×*; (**C**) 10*×*; and (**D**) 40*×*. In the context of initial autolytic phenomena consisting of detachment of the retinal epithelium from the choroid, vascular congestion phenomena (yellow arrows) and the presence of interspersed erythrocytes can be observed within the retinal layers (red arrows), with a particular representation affecting the inner nuclear layer (INL) and the outer nuclear layer (ONL).

**Figure 3:**
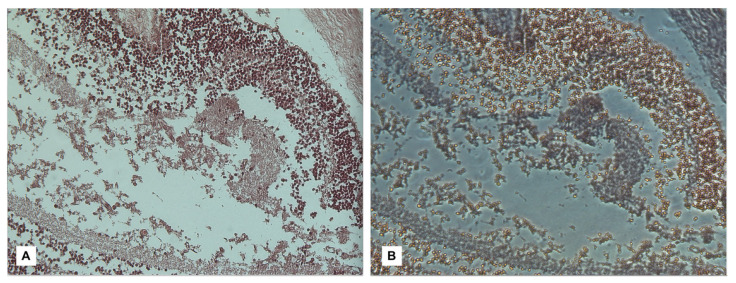
In reference to the subject of Figure 2, at a magnification of 40*×* with direct light (**A**), it is possible to associate the visualization in PCM (**B**), extremely effective in showing discoid-shaped erythrocytes within intra-retinal collections [98].

Furthermore, Maiese et al. [98] applied confocal spectral imaging (CSI) using Leica TCS SP2 equipment to such preparations, obtaining a brilliant result in visualizing the hemorrhagic area due to the peculiar spectral emission of hemoglobin compared to the surrounding tissues. However, it should be emphasized that non-innovative methods such as Phase Contrast Microscopy (PCM) represent a valid option for confirming the presence of free erythrocytes in examined samples due to their high sensitivity in highlighting the discoid shape of erythrocytes and enhancing them compared to the surrounding tissues (Figure 2 and Figure 3).

H&E staining is a basic method for the microscopic diagnosis of recent RHs, but also for other acute ocular hemorrhagic findings. Regarding ONSH, in fact, Puanglumyai et al. [71] have illustrated how this examination allows for an accurate visualization of extravascular erythrocytes in the epidural, subdural, and subarachnoid spaces as well as in the nerve fibers, allowing for an agile confirmation of autopsy findings or, alternatively, a new diagnosis in case of poor macroscopic evidence of hemorrhages. Oshima et al. [93], instead, used the same method for the detection of PSH, highlighting how these occurred precisely in correspondence with the Zinn–Haller arterial ring, supporting the pathogenetic mechanism proposed by the same author.

If the trauma has a sub-acute, remote, or chronic origin, the detection of RBCs in the sample may not be possible due to lysis phenomena. In addition to its diagnostic interest, histopathological investigation plays an important role in searching for elements useful in establishing the time interval between trauma and death due to the significant legal interest associated with the reconstruction of the exact moment when the alleged trauma occurred. Bais et al. [91], in this regard, started from the assumption that deposits of hemosiderin within retinal hemorrhagic foci are visible from 2 days after trauma, and that according to the British Child Abuse Working Party, deposits resulting from birth trauma can be observed up to about 3 months after birth [107]. However, in the absence of epidemiological studies on this issue, the author posed the question of what could be the maximum time for hemosiderin persistence in retinal tissues of AHT victims, establishing based on the results obtained that such residues can be observed for at least 32 months after the traumatic event, such a finding does not necessarily indicate chronicity of the trauma, and if such deposits exist in the context of frank hemorrhage, this does not exclude the possibility of recent trauma.

Indeed, as noted by Del Bigio and Phillips [94], a critical aspect of such investigations is related to the following factors:

- An uncomplicated vaginal birth and cesarian section are frequently related to microscopic hemorrhages in retina, orbital fat, and extra-ocular soft tissues for reasons unrelated to resuscitation attempts, but ONSH are exceptional findings in such cases;
- Water-soluble ferritin may be lost during routine histological processing techniques, leaving hemosiderin as the most represented compound.

Based on these precautionary considerations, the authors proceeded to study nine traumatic cases and 53 controls, highlighting in these cases a significantly higher rate of hemosiderin deposits in the subdural and subarachnoid spaces, as well as within the ON itself, in the intraorbital and intracanalicular portions. Regarding the post-traumatic interval (PTI), the team observed a minimum time of onset of 3 days and a maximum observability time extended up to 36 months after trauma.

However, these results do not constitute a validated dating system, but an attempt to set temporal limits within which to act, with the support of additional ancillary data. The team led by Delteil [95], instead, previously engaged with the construction of a staging system for SDH [76], applied a system based on three methods (H&E, histochemical iron staining with Perls Prussian Blue, and immunohistochemistry with CD68 immunolabeling) to a large case series (83 victims of AHT with a known PTI ranging from <12 h to 274 days), according to the following interpretive scheme (Table 7).

The results were validated by a statistical analysis conducted via a PTI chart shown in Table 8, which represents the only timing tool available nowadays. The scientific experience of Delteil et al. [95] has highlighted how, based on the means employed, a valid diagnostic aid is provided exclusively by the presence of these three elements: RBCs, siderophages, and retinal sclerosis. Significant differences were observed in the behavior over time of RHs compared to SDHs and SAHs, with coherence observed in only two aspects: the appearance of siderophages (PTI ≥ 3 days) and in the cellular and fibrous organization.

**Figure 4:**
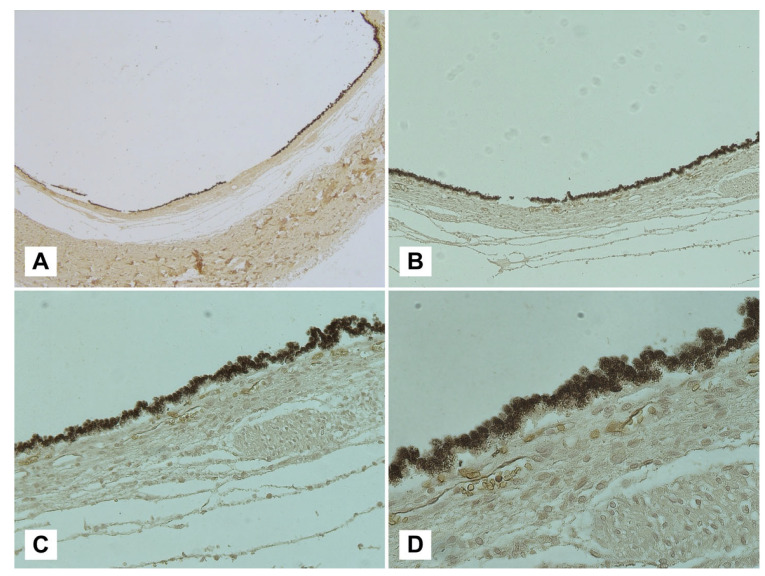
Visualization of a sub-retinal plane with glycophorin-A immunolabeling at different magnifications: (**A**) 2.5*×*; (**B**) 5*×*; (**C**) 10*×*; (**D**) 40*×*. Following the removal of the retinal membrane, a strong immunopositivity for the membrane antigen glycophorin-A can be observed at the level of the choroidal membrane, interpretable as sub-retinal blood collection.

Immunohistochemical analyses of samples, introduced in this paper through the work conducted by Delteil et al. [95], represent a frontier of modern histopathology recently applied in the field of RH. For this reason, the scientific evidence on the subject is still scarce, but undoubtedly useful for resolving cases of extraordinary complexity. Maiese et al. [98] used an erythrocyte-specific membrane sialoglycoprotein, glycophorin-A (CD235a), as an immunoreagent. This reagent, widely used in forensic practice as a marker of lesion vitality [108], is extremely useful in confirming the presence of a hemorrhagic area across retinal planes (Figure 4). However, the studies considered [98,99,100,101,102,103,104,105,106,107,108,109,110,111,112] do not address the issue of dating the lesions. Since a PTI of up to approximately 24 days has been established for the observation of intact or lysed RBCs through traditional H&E staining [95], further investigations could be useful in this regard.

The team os Bais [89], instead, applied immuno-histochemical methods already used for the diagnosis of severe axonal damage in the ophthalmic field. Both beta-amyloid precursor protein (β-APP) and ubiquitin are expressed within the central nervous system after mechanical trauma [29]; therefore, it was hypothesized that they could be used as markers of axonal injury (AI) affecting the retinal nerve fiber layer (NFL). In addition, anti-glial fibrillar acid protein (GFAP) antibodies were also used as a marker of reactive gliosis, a phenomenon linked to numerous pathological conditions (infections, ischemia, and Alzheimer’s disease) and also linked to cases of traumatic injury [30]. For this reason, the retina was analyzed by studying the individual layers (NFS; inner plexiform layer, IPL; INL; outer plexiform layer, OPL; ONL; and photoreceptor layer, PL) and sectors (periphery, equator, and posterior pole) and through the quantification of RH as slight, moderate, or extensive.

Regarding the optic nerve (ON), it was studied using beta-amyloid precursor protein (β-APP) immunolabeling and applying the Reichard et al. score, which quantifies it as slight and pinpoint, moderate, extensive, or widespread [113]. At the end of the study, it was possible to demonstrate the validity of β-APP and ubiquitin immunolabeling as markers of retinal axonal injury (RAI) in relation to AHT-related RH, and of β-APP alone as a marker of AI affecting the ON. Furthermore, the positivity of β-APP was directly correlated with the severity of ocular damage quantified by the topographic extension of the hemorrhage and the presence of vitreous and macular folds. β-APP and ubiquitin showed the highest positivity in the peripapillary region, probably due to anatomical and physiological reasons partly related to pediatric age and the mechanism of traumatic vitreoretinal and retinal-ON traction. From a temporal point of view, an interval of β-APP and ubiquitin positivity was observed from the time of presentation to healthcare professionals ranging from 2.5 h to 8 days. Finally, Bais et al. arrived at the following conclusions:

- β-APP and ubiquitin are valid markers for axonal and retinal damage and NO in cases of AHT, especially when used together;
- GFAP, on the other hand, did not show a statistically significant correlation with AHT;
- An RH extended to 20%–30% of the retinal area should be considered a strong indicator of AHT, as well as vitreal and orbital fat hemorrhage and macular folds.

A study of immunohistochemical markers for nerve damage in retinal tissue was also carried out by Bulirsch et al. [100]; in this case, the authors investigated the function of Müller cells, which are responsible for the structural, trophic, and homeostatic support of the retinal epithelium during fetal development. One of the immunological markers used was Nestin, for which an immunoreaction has been observed in AHT cases, but also in fetal and adult eyes. Consequently, it can be postulated that such results support the hypothesis of the regenerative potential of Müller cells. Another marker employed by Bulirsch et al. [100] was CD44, for which only AHT cases showed an immunoreaction (*p* < 0.05); lastly, GFAP immunopositivity has been found in AHT cases as well as in a premature infant [28] and after vaginal delivery. For these reasons, GFAP expression in suspected traumatic cases should be interpreted cautiously and considering all related findings. Immunological markers and results from Bulirsch et al. are summarized in Table 9.

Ultimately, the research conducted by Bulirsch et al. [100] can be considered a valuable attempt to apply new immunological markers to the field of AHT; however, the study design is excessively focused on the search for embryonic changes in the human eye, with an insufficient number of AHT cases to achieve statistical significance.

## 5. Conclusions

Since the association between RH and physical violence towards infants was discovered [2], the scientific knowledge has advanced to the extent that the diagnosis of even fatal AHT in cases dominated by anamnestic uncertainty and a scarcity of physical evidence is now possible.

The present systematic review aimed to clarify the scientific evidence derived from the most recent available literature, establishing an arbitrary interval corresponding to the last 6 years (2016–2022). This goal has been achieved, considering clinical, thanatological, radiological, autopsy, and histopathological aspects. Despite such an objective involving the inclusion of extremely heterogeneous evidence, a good degree of expositional and content homogeneity has been achieved, prioritizing forensic, innovative, and statistically analyzed content.

Despite the scarcity of content related to the clinical evaluation of the living subject, already supported by visualization and staging techniques validated by the scientific community, the scientific community is divided in establishing the diagnostic value of unilateral rather than bilateral retinal hemorrhages. However, current severity rating scales for RH [30,43] do not consider an emerging aspect related to the age stratification of AHT victims; based on recent evidence, there seems to be an anatomical predisposition in subjects <6 months of age to develop unilateral lesions, so such findings should be given particular weight in this age group [97].

Furthermore, it is emphasized that indirect ophthalmoscopy, burdened by a high operator-dependent variability, should always be integrated with second-level ophthalmological examinations such as DWFFP and OCT [88,99]. In post-mortem cases, due to the significant judicial interest involved, the use of non-invasive examinations (PMIO) [90] should be reserved for cases characterized by an extremely low suspicion, in which a primary ocular autopsy investigation would be excessively invasive and disfiguring. The only limitation, in this regard, is represented by the reduced time allowed by the advancement of sclero-corneal opacity phenomena.

Decisive progress, therefore, could be identified in the performance of targeted radiological investigations that, in a cephalic or total-body visualization context, allow the simultaneous search for bone fractures or brain parenchymal lesions. At present, the best method for studying the retina and its appendages (in particular the ON) is represented by MRI performed with specific protocols, i.e., GRE T2-w and 3D-SWI [80,96].

Knowing the specific lesion sites also allows for a more accurate autopsy search; in this context, enucleation of the orbital content through intracranial access is currently the preferred method, as it allows for the preservation and in situ observation of structures of fundamental diagnostic importance, namely the ON and periocular soft tissues [71,98]. The observation of macroscopic hemorrhagic alterations affecting these structures is, in fact, a predictive element of proven value, characterized by a significantly higher specificity than the retinal context [93,94].

Diagnostic confirmation, as well as the search for elements useful for dating the lesions, still finds a fundamental importance in histopathology. Among the most important contributions, it is possible to include the experience of Delteil et al. [95], who applied dating techniques tested on SDH to the ocular field, proving that the most important elements are represented by the presence of RBCs (immediate appearance and cytolysis after the first 24 h), siderophagic cells (after a PTI of 3 days), and retinal sclerosis or ON atrophy (after 1 week).

Regarding the application of immunohistochemical methods in ocular studies, it is possible to include glicophoryn-A [98], a membrane antigen of RBCs, as a marker of intratissue hemorrhagic extravasation particularly useful in cases of poor erythrocyte representation or lysis of erythrocytes in the absence of early hemosiderin deposits visible through Perls’ histochromatic staining. Attempts at further immunolabeling of retinal or optic nerve tissue currently have promising results, but are not yet equipped with sufficient diagnostic or temporal value for use in daily forensic practice.

**Figure 5:**
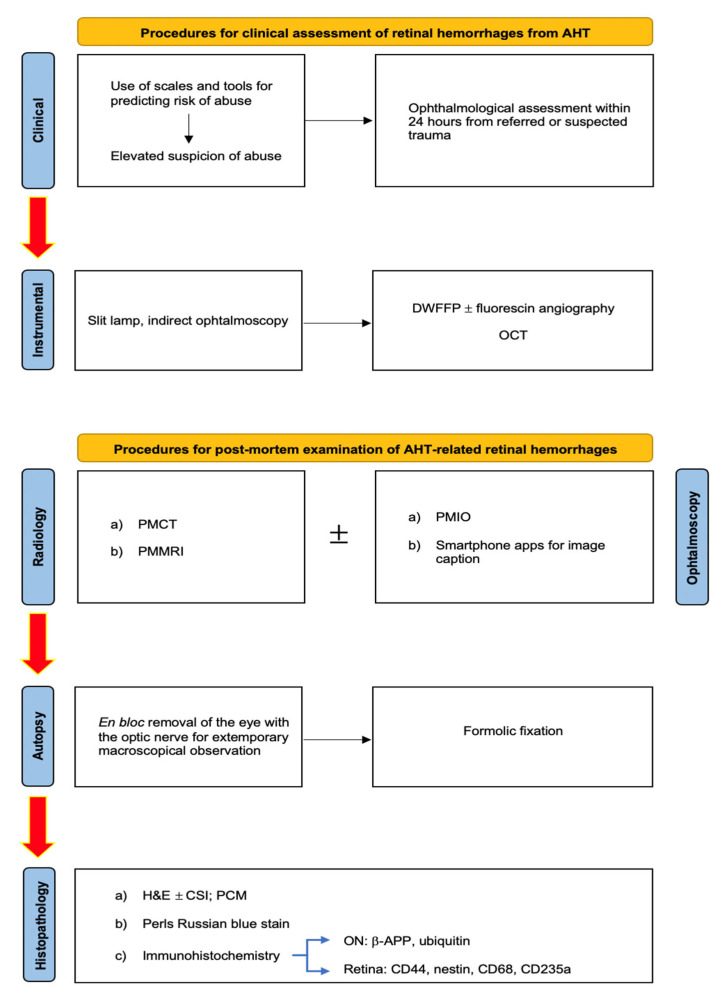
Operative framework for clinical and forensic assessment of AHT-related RHs.

At the end of this work, it is possible to present an operational framework aimed at the daily application of the currently available scientific evidence to assist in the resolution of cases of judicial interest; therefore, the following diagram exposes the actual state-of-the-art methods in diagnosing and the timing of AHT-related retinal and optical nerve injuries (Figure 5).

## Figures and Tables

**Table 1:** Main AHT-related ocular findings and severity scale [44].

Retina	
*RH Type*	*Main Findings*
Mild	Few in number, intraretinal, confined to posterior pole
Moderate	Numerous, multi-layered, extended over ora serrata
Severe	Same aspects of moderate one, but bilateral
Other aspects	Macular folds, emovitreus
**Other ocular structures**	
*Anatomical district*	*Main findings*
Cornea	Abrasions, lacerations, cloudings
Crystalline lens	Dislocations, damage to suspensory ligaments of lens, ciliar body and muscle
Conjunctiva	Subjunctival hemorrhages
Optical nerve sheaths	Hemorrhage, most frequently subdural
Periorbital adipose tissue and extraocular muscles	Hemorrhage

**Table 2:** Main non-AHT-related conditions causing RH.

Non-AHT Conditions Related to RH	Incidence	Timing	Risk Factors/Positive Elements	Mechanism	Morphologic Aspect	Exclusion Elements
Birth trauma	Approximately one-third of newborns [38]	First two days of life, 85% of cases heal within 2 weeks [79,80]	Vacuum-assisted delivery [31]	Perinatal hemodynamic changes, ocular compression, prostaglandin release	Often numerous, extended over ora serrata (such as AHT-related) but only intraretinal, with rare retinal folds	RH with numerous, diffuse, extraretinal, duration extended over the first month of life
Accidental head injury	Less than 4% according to multiple authors; short falls have an RH incidence close to 0% [36,81,82,83]	Same as AHT	Unambiguous and consistent history given by parents, presence of witnesses, other lesions compatible with referred kind and force of impact	Direct impact, Terson’s Syndrome, rapid acceleration and rotational movements of the head	Often confined to posterior pole, few in number, rarely subretinal. Severe accidents or impacts may determine extended lesions [84]	Absence of other lesions, suspicious behavior and history given by parents, absence of witnesses
Raised ICP	Not estimated	Same as AHT	Severe elevation of ICP	Terson’s Syndrome	Superficial, intraretinal, located on or close to the optic disc [84]	Absence of papilledema (present in only 10% cases of AHT), other different patterns
Systemic diseases	Variable	Coagulopathy (leukemia, thrombocytopenia, severe anemia, Vitamin K and factors deficiencies, and hemolytic uremic syndrome); raised ICP (glutaric aciduria type 1, meningitis); thrombosis of retinal artery (e.g., endocarditis), damage to the retinal endothelium (e.g., vasculitis)	Related to specific pathology	Deficiency of coagulation mechanisms	Low number and extension	Lack of diagnosis from accurate clinical and laboratory assessments

**Table 3:** Main characteristics and technical differences among selected studies.

Paper	Year	Country	Category	Aim	Number of Subjects	Age Range of the Studied Population	Methodology	Specific Investigations/Reactants	Statistical Analysis and Validation	Limitations
Bais et al. [89]	2016	The Netherlands	Retrospective cross-sectional study	Investigation of new markers as diagnostic tools for discrimination between AHT and non-AHT	37 deceased infants (21 AHT cases, 16 controls)	10 to 1041 days	Immunohistochemistry	β-APP, ubiquitin, GFAP	Yes	Need for further experimentation for international validation
Lantz et al. [90]	2016	USA	Technical note	Description of smartphone still-image capture techniques in PMIO	Not applicable	Not applicable	Employment of smartphone flashlight and camera in addition to traditional PMIO	Not applicable	No	First and only article on the subject
Bais et al. [91]	2016	The Netherlands	Case report	Description of a long-term hemosiderin persistence in an AHT case	1 deceased infant, prior victim of AHT	5 months	Histopathology	Hematoxylin and eosin (H&E); Perl’s iron stain	No	Absence of a large cohort
Fieß et al. [88]	2016	Germany	Case report	Illustration of the correct process to diagnose AHT from eye examinations	1 infant	10 months	Clinical examination, autopsy, histopathology	Ophtalmoscopy and histological confirmation by H&E staining	No	Absence of a large cohort, no statistical validation
Yusuf et al. [92]	2017	United Kingdom	Case series	Audit the use of non-contact ultra-widefield retinal imaging in infants with suspected abusive head trauma (AHT)	5 infants	1 to 15 months	Clinical assessment in living subjects	P200MA Scanning Laser Ophthalmoscope	No	Absence of a large cohort, no statistical validation
Puanglumyai et al. [71]	2017	Thailand	Case series	Demonstration of ONSH importance over RH in AHT diagnosis	11 deceased infants, victims of AHT	1 to 24 months	Autopsy, histopathology	Removal of orbital roof, bilateral exposure and fixation of ONs, H&E staining	No	Absence of a large cohort, no statistical validation
Oshima et al. [93]	2017	Japan	Case report	Investigating the mechanism of peripapillary scleral hemorrhages	4 subjects. of whom 1 infant was the victim of AHT	1 month	Autopsy, histopathology	Microscopic observation of peripapillary scleral hemorrhages	No	Simple description of an encountered case
Del Bigio et al. [94]	2017	Canada	Retrospective monocentric study	Creating an injury timing interval chart based on microscopic findings	62 infants, of whom 9 were victims of AHT	8 to 124 weeks	Autopsy, histopathology	Hematoxylin and eosin (H&E); Perl’s iron stain; Martius scarlet blue	Yes	Absence of complete ocular examination
Delteil et al. [95]	2019	France	Retrospective multicenter study	Construction of a histological diagnostic tool for RH timing	83 deceased infants (48 males, 35 females), victims of AHT	0 to 36 months	Histopathology	Hematoxylin and eosin (H&E); Perl’s iron stain	Yes	-
Zuccoli et al. [96]	2020	Italy	Retrospective monocentric study	Providing ONSH evidence in survival AHT cases	13 infant victims of AHT	1.2 to 20.8 months	MRI	3D-SWI protocol	Yes	Absence of control subjects; lack of intracranial findings analysis
Barth et al. [87]	2020	Germany	Case report	Highlighting the possibility of unilateral RHs in confirmed AHT cases	2 infants	2 and 2.5 months	Clinical assessment in living subjects	Ophthalmoscopy	No	Anecdotal validity, despite growing evidence in literature of unilateral RHs among AHT victims
Cartocci et al. [78]	2020	Italy	Review	Broad view on the main MRI findings in the central nervous system in cases of AHT	Not applicable	Not applicable	MRI	Combination of coronal T2-w, T1-w and axial GRE T2-w protocols to detect extra-axial hemorrhages such RHs	No	Lack of large-cohort studies available at the time; variability in techniques across considered studies
Maiese et al. [98]	2021	Italy	Review	New immunohistochemical technique proposal	Not applicable	Not applicable	Histopathology	Application of Glycophorin A immunoreaction and confocal laser scanning microscopy to RHs	No	Need for large scale validation of the proposed technique
Oliva et al. [99]	2021	Italy	Retrospective monocentric study	Combination of RETcam and OCT as a diagnostic and prognostic tool in AHT victims	6 infants	0.6 to 10 months	Clinical assessment in living subjects	Combination of RETcam and OCT	Yes	Need for further application of the proposed protocol
Bulirsch et al. [100]	2021	Germany	Prospective cross-sectional study	Investigation of retinal Müller cells immunoreactivity in response to AHT in children	37 infants, of whom 5 were victims of AHT	1 to 8 months	Histopathology	Hematoxylin and eosin (H&E); Periodic-acid Schiff (PAS) reaction; Immunoreaction against Nestin, CD44, collagen type IX, GFAP	Yes	First article on the subject, need for a large cohort
Moskwa et al. [97]	2022	France	Retrospective monocentric study	Strengthening the correlation between specific ophthalmological lesions and AHT	133 infants	14 days to 10 months	Clinical assessment in living subjects	Ophthalmoscopy	Yes	Need for a multicentric data collection and analysis of other variables

**Table 4:** Critical assessment of the quality of the selected manuscripts according to Newcastle–Ottawa scale: cohort and case control studies [101].

	Selection (Star Number)	Comparability (Star Number)	Outcome (Star Number)
Bais et al. [89]	3	1	3
Puanglumyai et al. [71]	2	Not applicable ^†^	2
Del Bigio et al. [94]	4	1	2
Delteil et al. [95]	2	Not applicable ^†^	2
Zuccoli et al. [96]	1	Not applicable ^†^	3
Moskwa et al. [97]	3	Not applicable ^†^	2
Oliva et al. [99]	1	Not applicable ^†^	3
Bulirsch et al. [100]	2	1	3

^†^ Comparability method not applicable in cohort studies.

**Table 5:** Critical assessment of the quality of the selected manuscripts according to the JBI Critical Appraisal checklist for case reports [102].

	Barth et al. [87]	Fieß et al. [88]	Bais et al. [91]	Yusuf et al. [92]	Oshima et al. [93]
Were patient’s demographic characteristics clearly described?	Unclear	Yes	Yes	Unclear	Unclear
Was the patient’s history clearly described and presented as a timeline?	Unclear	Yes	Yes	Yes	Unclear
Was the current clinical condition of the patient on presentation clearly described?	Yes	Yes	Yes	Yes	Yes
Were diagnostic tests or assessment methods and the results clearly described?	Yes	Yes	Yes	Yes	Yes
Was the intervention(s) or treatment procedure(s) clearly described?	Yes	Yes	Yes	Yes	Yes
Was the post-intervention clinical condition clearly described?	Not applicable	Not applicable	Not applicable	Not applicable	Not applicable
Were adverse events (harms) or unanticipated events identified and described?	Not applicable	Not applicable	Not applicable	Not applicable	Not applicable
Does the case report provide takeaway lessons?	Yes	Yes	Yes	Yes	Yes

**Table 6:** Critical assessment of the quality of the selected manuscripts according to the JBI Critical Appraisal checklist for systematic reviews [103].

	Cartocci et al. [78]	Maiese et al. [98]
Is the review question clearly and explicitly stated?	Yes	Yes
Were the inclusion criteria appropriate for the review question?	Yes	Yes
Was the search strategy appropriate?	Unclear	Yes
Were the sources and resources used to search for studies adequate?	Yes	Yes
Were the criteria for appraising studies appropriate?	Yes	Yes
Was critical appraisal conducted by two or more reviewers independently?	No	Yes
Were there methods to minimize errors in data extraction?	No	No
Were the methods used to combine studies appropriate?	Unclear	Yes
Was the likelihood of publication bias assessed?	No	No
Were recommendations for policy and/or practice supported by the reported data?	Yes	Yes
Were the specific directives for new research appropriate?	Yes	Yes

**Table 7:** Methodological analysis modified from Delteil et al. [95].

Item	Technique	Morphology/Quantification	Attributed PTI	Notes
RBCs	H&E	Intact	First 24 h	Differentiation from autolytic mechanisms by comparison with other structures
		Lysed	From 2 to 24 days	
		Mixed		
Fibrin/platelets	H&E	Presence/Absence	First 72 h	-
Leukocytes	H&E	PMNs and/or lymphocytes	PMNs from 12 h to 17 days (peak within 48–96 h); lymphocytes from 12 h to 23 days (peak within 72–96 h)	-
Macrophages	CD68 immunolabeling	200× magnification field count for semi-quantitative study	From 72 h (minimal PTI observed: 12 h)	Cells per filed proposed: 0–3
Hemoglobin degradation	Perls Prussian blue stain	Detection of siderophages and/or hematoid deposits within affected tissue	From 72 h	Semi-quantitative classification as absent, early, moderate, or abundant
Neovascularization	H&E	Absent/present	From the 6th to the 70th day	Grades of presence: capillary proliferation, giant capillaries, arterioles
Fibrous organization	H&E	Absent/present	From the 2nd week	Aspects of retinal sclerosis or ON atrophy

**Table 8:** Reconstruction of PTI based on histopathological features of RH (modified from Delteil et al. [95]).

Estimated PTI	Microscopic Alterations
0–3 days	Organization of fibrin and platelets inside hemorrhage
From the 2nd day	Lysis of RBCs
From the 6th hour to the 23rd day	Lymphocytes migration inside RH
From the 12th hour to the 17th day	Macrophages migration inside RH
From the 4th day	Siderophages inside RH; fibrous organization with fibroblasts inside RH
From the 4th day to 1 month	Collagen deposition
From the 7th day	Neovascularization
From the 8th day	Retinal sclerosis

**Table 9:** Synopsis of immunoreactants employed by Bulirsch et al. to understand Müller cell alteration in AHT and relative results [100].

Immunoreactants	Function	Results
Nestin	Marker of stem cell regeneration, intermediate filaments production	Immunoreaction observed in AHT cases, but also in fetal and adult eyes.
CD44	Marker of stem cell regeneration	High specificity for AHT cases.
GFAP	Intermediate filaments production, reactive glia cells activation	Immunopositivity found in AHT cases as well as in a premature infant.

## Data Availability

All data supporting the reported results can be found in the manuscript.

## References

[1] Rosén M., Lynøe N., Elinder G., Hallberg B., Sundgren P., Eriksson A. (2017). Shaken baby syndrome and the risk of losing scientific scrutiny. Acta Paediatr..

[2] Tardieu A. (1860). Étude Médico-légale sur les sévices et mauvais traitements exercés sur des enfants. Ann. Hyg. Publique Med. Leg..

[3] Caffey J. (1972). On the theory and practice of shaking infants. Its potential residual effects of permanent brain damage and mental retardation. Am. J. Dis. Child..

[4] Duhaime A.C., Gennarelli T.A., Thibault L.E., Bruce D.A., Margulies S.S., Wiser R. (1987). The shaken baby syndrome. A clinical, pathological, and biomechanical study. J. Neurosurg..

[5] Minns R.A., Jones P.A., Tandon A., Fleck B.W., Mulvihill A.O., Elton R.A. (2012). Prediction of inflicted brain injury in infants and children using retinal imaging. Pediatrics.

[6] Fujiwara T., Okuyama M., Miyasaka M. (2008). Characteristics that distinguish abusive from non-abusive head trauma among young children who underwent head computed tomography in Japan. Pediatrics.

[7] Myhre M., Grøgaard J., Dyb G., Sandvik L., Nordhov M. (2007). Traumatic head injury in infants and toddlers. Acta Paediatr..

[8] Tung G.A., Kumar M., Richardson R.C., Jenny C., Brown W.D. (2006). Comparison of accidental and nonaccidental traumatic head injury in children on non-contrast computed tomography. Pediatrics.

[9] Vinchon M., Defoort Dhellemmes S., Desurmont M., Dhellemmes P. (2005). Accidental and nonaccidental head injuries in infants: A prospective study. J. Neurosurg..

[10] Keenan H.T., Runyan D.K., Marshall S.W., Nocera M.A., Merten D.F. (2004). A Population-Based Comparison of Clinical and Outcome Characteristics of Young Children With Serious Inflicted and Noninflicted Traumatic Brain Injury. Pediatrics.

[11] Bechtel K., Stoessel K., Leventhal J.M., Ogle E., Teague B., Lavietes S., Banyas B., Allen K., Dziura J., Duncan C. (2004). Characteristics that distinguish accidental from abusive injury in hospitalized young children with head trauma. Pediatrics.

[12] Harding B., Risdon R.A., Krous H.F. (2004). Shaken baby syndrome. BMJ.

[13] Christian C.W., Block R., Committee on Child Abuse and Neglect, American Academy of Pediatrics (2009). Abusive head trauma in infants and children. Pediatrics.

[14] Parks S.E., Annest J.L., Hill H.A., Karch D.L. (2012). Pediatric Abusive Head Trauma: Recommended Definitions for Public Health Surveillance and Research.

[15] Leetch A.N., Woolridge D. (2013). Emergency Department Evaluation of Child Abuse. Emerg. Med. Clin. N. Am..

[16] Lopes N.R., Eisenstein E., Williams L.C. (2013). Abusive head trauma in children: A literature review. J Pediatr..

[17] Graupman P., Winston K.R. (2006). Nonaccidental head trauma as a cause of childhood death. J. Neurosurg. Pediatr..

[18] Kesler H., Dias M.S., Shaffer M., Rottmund C., Cappos K., Thomas N.J. (2008). Demographics of abusive head trauma in the Commonwealth of Pennsylvania. J. Neurosurg. Pediatr..

[19] Parmar C.D., Sinha A.K., Hayhurst C., May P.L., O’Brien D.F. (2007). Epidural hematoma formation following trivial head trauma in a child with osteogenesis imperfecta. J. Neurosurg. Pediatr..

[20] Duhaime A.C. (2008). Demographics of abusive head trauma. J. Neurosurg. Pediatr..

[21] Starling S.P., Patel S., Burke B.L., Sirotnak A.P., Stronks S., Rosquist P. (2004). Analysis of Perpetrator Admissions to Inflicted Traumatic Brain Injury in Children. Arch. Pediatr. Adolesc. Med..

[22] Starling S.P., Holden J.R., Jenny C. (1995). Abusive Head Trauma: The Relationship of Perpetrators to Their Victims. Pediatrics.

[23] Wu S.S., Ma C.-X., Carter R.L., Ariet M., Feaver E.A., Resnick M.B., Roth J. (2004). Risk factors for infant maltreatment: A population-based study. Child Abus. Negl..

[24] Wygnanski-Jaffe T., Morad Y., Levin A.V. (2009). Pathology of retinal hemorrhage in abusive head trauma. Forensic Sci. Med. Pathol..

[25] Babl F.E., Pfeiffer H., Kelly P., Dalziel S.R., Oakley E., Borland M.L., Kochar A., Dalton S., Cheek J.A., Gilhotra Y. (2019). Paediatric abusive head trauma in the emergency department: A multicentre prospective cohort study. J. Paediatr. Child Health.

[26] Greeley C.S. (2015). Abusive Head Trauma: A Review of the Evidence Base. Am. J. Roentgenol..

[27] Geddes J.F., Hackshaw A.K., Vowles G.H., Whitwell H.L. (2001). Neuropathology of inflicted head injury in children. I. Patterns of brain damage. Brain.

[28] Morad Y., Kim Y.M., Armstrong D.C., Huyer D., Mian M., Levine A.V. (2002). Correlation between retinal abnormalities and intracranial abnormalities in the shaken baby syndrome. Am. J. Ophthalmol..

[29] Hymel K.P., Karst W., Marinello M., Herman B.E., Frazier T.N., Carroll C.L., Armijo-Garcia V., Musick M., Weeks K., Haney S.B. (2022). Pediatric Brain Injury Research Network (PediBIRN) Investigators. Screening for pediatric abusive head trauma: Are three variables enough?. Child Abus. Negl..

[30] Kanya Iyer A., Lemos N.P. (2019). Are we looking for retinal haemorrhages?. Med. Sci. Law.

[31] La Russa R., Maiese A., Cipolloni L., Di Fazio N., Delogu G., De Matteis A., Del Fante Z., Manetti F., Frati P., Fineschi V. (2022). Diagnostic assessment of traumatic brain injury by vacuum extraction in newborns: Overview on forensic perspectives and proposal of operating procedures. Front. Biosci..

[32] Squier W. (2011). The “Shaken Baby” syndrome: Pathology and mechanisms. Acta Neuropathol..

[33] Piteau S.J., Ward M.G., Barrowman N.J., Plint A.C. (2012). Clinical and radio-graphic characteristics associated with abusive and nonabusive head trauma: A systematic review. Pediatrics.

[34] Elinder G., Eriksson A., Hallberg B., Lynøe N., Sundgren P.M., Rosén M., Engström I., Erlandsson B.E. (2018). Traumatic shaking: The role of the triad in medical investigations of suspected traumatic shaking. Acta Paediatr..

[35] McKeag H., Christian C.W., Rubin D., Daymont C., Pollock A.N., Wood J. (2013). Subdural hemorrhage in pediatric patients with enlargement of the sub arachnoid spaces. J. Neurosurg. Pediatr..

[36] Bhardwaj G., Chowdhury V., Jacobs M.B., Moran K.T., Martin F.J., Coronet M.T. (2010). A systematic review of the diagnostic accuracy of ocular signs in pediatric abusive head trauma. Ophthalmology.

[37] Kivlin J.D., Simons K.B., Lazoritz S., Ruttum M.S. (2000). Shaken baby syndrome. Ophthalmology.

[38] Watts P., Maguire S., Kwok T., Talabani B., Mann M., Wiener J., Lawson Z., Kemp A. (2013). Newborn retinal hemor-rhages: A systematic review. J. AAPOS.

[39] Levin A.V. (2010). Retinal hemorrhage in abusive head trauma. Pediatrics.

[40] Levinson J.D., Pasquale M.A., Lambert S.R. (2016). Diffuse bilateral retinal hemorrhages in an infant with a coagulopathy and prolonged cardiopulmonary resuscitation. J. AAPOS.

[41] Rivera F. (1993). Population-based study of fall injuries in children and adolescents resulting in hospitalization or death. Pediatrics.

[42] Chadwick D.L., Bertocci G., Castillo E., Frasier L., Guenther E., Hansen K., Herman B., Krous H.F. (2008). Annual Risk of Death Resulting from Short Falls Among Young Children: Less Than 1 in 1 Million. Pediatrics.

[43] Binenbaum G., Forbes B.J. (2014). The eye in child abuse: Key points on retinal hemorrhages and abusive head trauma. Pediatr. Radiol..

[44] Terson P.D.A. (1900). Hemorrhage in the vitreous body during cerebral hemorrhage. La Clin. Ophthalmol..

[45] Muller P.J., Deck J.H.N. (1974). Intraocular and optic nerve sheath hemorrhage in cases of sudden intracranial hypertension. J. Neurosurg..

[46] Koto T., Takubo K., Ishida S., Shinoda H., Inoue M., Tsubota K., Okada Y., Ikeda E. (2007). Hypoxia Disrupts the Barrier Function of Neural Blood Vessels through Changes in the Expression of Claudin-5 in Endothelial Cells. Am. J. Pathol..

[47] Firsching R., Muller C., Pauli S.U., Voellger B., Rohl F.W., BehrensBaumann W. (2011). Noninvasive assessment of intracranial pressure with venous ophthalmodyna mometry. Clinical article. J. Neurosurg..

[48] Lantz P.E., Carlson J., Mott R. Extensive Hemorrhagic Retinopathy, Perimacular Retinal Fold, Retinoschisis, and Retinal Hemorrhage Progression Associated with a Fatal Spontaneous, Non-Traumatic, Intracranial Hemorrhage in an Infant. (Abstract presented 21 February 2013 at the Am. Acad. Forens. Sci. Annual Meeting, Washington, DC). http://www.aafs.org/sites/default/files/pdf/ProceedingsWashingtonDC2013.pdf.

[49] Levin A.V., Christian C.W., Committee on Child Abuse and Neglect, Section on Ophthalmology (2010). The eye exam ination in the evaluation of child abuse. Pediatrics.

[50] Coats B., Binenbaum G., Peiffer R.L., Forbes B.J., Margulies S.S. (2010). Ocular hemorrhages in neonatal porcine eyes from single, rapid rotational events. Investig. Ophthalmol. Vis. Sci..

[51] Gabaeff S.C. (2016). Exploring the controversy in child abuse pediatrics and false accusations of abuse. Leg. Med..

[52] Abed Alnabi W., Tang G.J., Eagle R.C., Gulino S., Thau A., Levin A.V. (2019). Pathology of perimacular folds due to vitreoretinal traction in abusive head trauma. Retina.

[53] Mills M. (1998). Funduscopic lesions associated with mortality in shaken baby syndrome. J. AAPOS.

[54] Binenbaum G., Christian C.W., Ichord R.N., Ying G.-S., Simon M.A., Romero K., Pollock A.N., Forbes B.J. (2013). Retinal hemorrhage and brain injury patterns on diffusion-weighted magnetic resonance imaging in children with head trauma. J. Am. Assoc. Pediatr. Ophthalmol. Strabismus.

[55] Reynolds J.D., Olitsky S.E. (2010). Pediatric Retina.

[56] Kellogg N.D. (2007). American Academy of Pediatrics Committee on Child Abuse and Neglect. Evaluation of suspected child physical abuse. Pediatrics.

[57] Vinchon M., de Foort-Dhellemmes S., Desurmont M., Delestret I. (2010). Confessed abuse versus witnessed accidents in infants: Comparison of clinical, radiological, and ophthalmological data in corroborated cases. Child’s Nerv. Syst..

[58] Högberg G., Colville-Ebeling B., Högberg U., Aspelin P. (2016). Circularity bias in abusive head trauma studies could be diminished with a new ranking scale. Egypt. J. Forensic Sci..

[59] Ewing-Cobbs L., Prasad M., Kramer L., Louis P.T., Baumgartner J., Fletcher J.M., Alpert B. (2000). Acute neuroradiological findings in young children with inflicted or noninflicted traumatic brain injury. Child’s Nerv. Syst..

[60] Binenbaum G., Forbes B.J., Reghupathi R., Judkins A., Rorke L., Margulies S.S. (2007). An animal model to study retinal hemorrhages in nonimpact brain injury. J. Am. Assoc. Pediatr. Ophthalmol. Strabismus.

[61] Serbanescu I., Brown S.M., Ramsay D., Levin A.V. (2008). Natural animal shaking: A model for non-accidental head injury in children?. Eye.

[62] Coats B., Binenbaum G., Smith C., Peiffer R.L., Christian C.W., Duhaime A.-C., Margulies S.S., Shuman M.J., Hutchins K.D., Reynolds B.B. (2016). Cyclic head rotations produce modest brain injury in infant piglets. J. Neurotrauma.

[63] Glowinski S., Majdanik S., Glowinska A., Majdanik E. (2021). Trauma in a shaken infant? A case study. Aggress. Violent Behav..

[64] Gabaeff S.C. (2011). Challenging the Pathophysiologic Connection between Subdural Hematoma, Retinal Hemorrhage and Shaken Baby Syndrome. WestJEM 21.2 March Issue.

[65] Lynøe N., Elinder G., Hallberg B., Rosén M., Sundgren P., Eriksson A. (2017). Insufficient evidence for ‘shaken baby syndrome’—A systematic review. Acta Paediatr..

[66] Strouse P.J. (2018). Shaken baby syndrome is real. Pediatr. Radiol..

[67] Lantz P.E., Adams G.G.W. (2005). Postmortem Monocular Indirect Ophthalmoscopy. J. Forensic Sci..

[68] Emerson M.V., Jakobs E., Green W.R. (2007). Ocular autopsy and histopathologic features of child abuse. Ophthalmology.

[69] Wygnanski-Jaffe T., Levin A.V., Shafiq A., Smith C., Enzenauer R.W., Elder J.E., Morin J.D., Stephens D., Atenafu E. (2006). Postmortem Orbital Findings in Shaken Baby Syndrome. Am. J. Ophthalmol..

[70] Gnanaraj L., Gilliland M.G.F., Yahya R.R., Rutka J.T., Drake J., Dirks P., Levin A.V. (2005). Ocular manifestations of crush head injury in children. Eye.

[71] Puanglumyai S., Lekawanvijit S. (2017). The importance of optic nerve sheath hemorrhage as a postmortem finding in cases of fatal abusive head trauma: A 13-year study in a tertiary hospital. Forensic Sci. Int..

[72] Migueis G.F.J., Fernandes F.A.O., Ptak M., Ratajczak M., Alves de Sousa R.J. (2019). Detection of bridging veins rupture and subdural haematoma onset using a finite element head model. Clin. Biomech..

[73] Murphy S., Thomas N.J., Gertz S.J., Beca J., Luther J.F., Bell M.J., Wisniewski S.R., Hartman A.L., Tasker R.C., Investigators of the Approaches and Decisions in Acute Pediatric Traumatic Brain Injury (ADAPT) Study (2017). Tripartite stratification of the Glasgow Coma Scale in children with severe traumatic brain injury and mortality: An analysis from a multi-center comparative effectiveness study. J. Neurotrauma..

[74] Riffenburgh R.S. (2005). Ocular hemorrhage in autopsies of child abuse victims. Clin. Surg. Opthalmol..

[75] Kodikara S., Pollanen M. (2012). Fatal pediatric head injury due to toppled television: Does the injury pattern overlap with abusive head trauma?. Leg. Med..

[76] Budenz D.L., Farber M.G., Mirchandani H.G., Park H., Rorke L.B. (1994). Ocular and Optic Nerve Hemorrhages in Abused Infants with Intracranial Injuries. Ophthalmology.

[77] Altinok D., Saleem S., Zhang Z., Markman L., Smith W. (2009). MR imaging findings of retinal hemorrhage in a case of nonaccidental trauma. Pediatr. Radiol..

[78] Cartocci G., Fineschi V., Padovano M., Scopetti M., Rossi-Espagnet M.C., Giannì C. (2021). Shaken Baby Syndrome: Magnetic Resonance Imaging Features in Abusive Head Trauma. Brain Sci..

[79] Hughes L.A., May K., Talbot J.F., Parsons M.A. (2006). Incidence, distribution, and duration of birth-related retinal hemorrhages: A prospective study. J. AAPOS.

[80] Binenbaum G., Mirza-George N., Christian C.W., Forbes B.J. (2009). Odds of abuse associated with retinal hemorrhages in children suspected of child abuse. J. AAPOS.

[81] Maguire S.A., Watts P.O., Shaw A.D., Holden S., Taylor R.H., Watkins W.J., Mann M.K., Tempest V., Kemp A.M. (2012). Retinal haemorrhages and related findings in abusive and non-abusive head trauma: A systematic review. Eye.

[82] Sturm V., Knecht P.B., Landau K., Menke M.N. (2008). Rare retinal haemorrhages in translational accidental head trauma in children. Eye.

[83] Kivlin J.D., Currie M.L., Greenbaum V.J., Simons K.B., Jentzen J. (2008). Retinal hemorrhages in children following fatal motor vehicle crashes: A case series. Arch. Ophthalmol..

[84] Binenbaum G., Rogers D.L., Forbes B.J., Levin A.V., Clark S.A., Christian C.W., Liu G.T., Avery R. (2013). Patterns of Retinal Hemorrhage Associated With Increased Intracranial Pressure in Children. Pediatrics.

[85] Shamseer L., Moher D., Clarke M., Ghersi D., Liberati A., Petticrew M., Shekelle P., Stewart L.A., PRISMA-P Group (2015). Preferred reporting items for systematic review and meta-analysis protocols (PRISMA-P) 2015: Elaboration and explanation. BMJ.

[86] Richardson W.S., Wilson M.C., Nishikawa J., Hayward R.S. (1995). The well-built clinical question: A key to evidence-based decisions. ACP J. Club.

[87] Barth T., Altmann M., Batzlsperger C., Jägle H., Helbig H. (2020). Unilaterale Netzhautblutungen bei Säuglingen—2 Fälle von Schütteltrauma? [Unilateral retinal hemorrhage in infants-two cases of shaken baby syndrome?]. Ophthalmologe.

[88] Fieß A., Dithmar S., Kölb-Keerl R., Kunze A., Riße M., Knuf M., Bauer J. (2016). Retinale Blutungen und venöse Stase bei einem 10 Monate alten Säugling nach Sturz? [Retinal bleeding and venous stasis in a 10-month-old infant after a fall?]. Ophthalmologe.

[89] Bais B., Kubat B., Motazedi E., Verdijk R.M. (2015). β-Amyloid Precursor Protein and Ubiquitin Immunohistochemistry Aid in the Evaluation of Infant Autopsy Eyes with Abusive Head Trauma. Am. J. Ophthalmol..

[90] Lantz P.E., Schoppe C.H., Thibault K.L., Porter W.T. (2015). Smartphone Image Acquisition During Postmortem Monocular Indirect Ophthalmoscopy. J. Forensic Sci..

[91] Bais B., Karst W.A., Kubat B., Verdijk R.M. (2016). Persistent Retinal Iron in Abusive Head Trauma. J. Forensic Sci..

[92] Yusuf I.H., Barnes J.K., Fung T.H.M., Elston J.S., Patel C.K. (2017). Non-contact ultra-widefield retinal imaging of infants with suspected abusive head trauma. Eye.

[93] Oshima T., Yoshikawa H., Koda Y., Ohtani M., Tsukamoto S., Mimasaka S. (2017). Four intracranial injury cases with peripapillary scleral hemorrhage—Reconsidering the mechanism of hemorrhage. Leg. Med..

[94] Del Bigio M.R., Phillips S.M. (2017). Retroocular and Subdural Hemorrhage or Hemosiderin Deposits in Pediatric Autopsies. J. Neuropathol. Exp. Neurol..

[95] Delteil C., Kolopp M., Capuani C., Humez S., Boucekine M., Leonetti G., Torrents J., Tuchtan L., Piercecchi M.-D. (2019). Histological dating of subarachnoid hemorrhage and retinal hemorrhage in infants. Forensic Sci. Int..

[96] Zuccoli G. (2021). Novel in vivo depiction of optic nerves hemorrhages in child abuse: A 3D-SWI pilot study. Neuroradiology.

[97] Moskwa R., Todeschi J., Wiedemann-Fode A., Stella I., Joud A., Klein O. (2022). Ophthalmological lesions in shaken baby syndrome: A retrospective analysis of 133 consecutive cases (1992–2018). Neurochirurgie.

[98] Maiese A., Iannaccone F., Scatena A., Del Fante Z., Oliva A., Frati P., Fineschi V. (2021). Pediatric Abusive Head Trauma: A Systematic Review. Diagnostics.

[99] Oliva A., Grassi S., Cazzato F., Jabbehdari S., Mensi L., Amorelli G., Orazi L., Arena V., Lepore D. (2022). The role of retinal imaging in the management of abusive head trauma cases. Int. J. Legal Med..

[100] Bulirsch L.M., Loeffler K.U., Holz F.G., Koinzer S., Nadal J., Müller A.M., Herwig-Carl M.C. (2022). Spatial and temporal immunoreaction of nestin, CD44, collagen IX and GFAP in human retinal Müller cells in the developing fetal eye. Exp. Eye Res..

[101] Wells G.A., Shea B., O’Connell D., Peterson J., Welch V., Losos M., Tugwell P. The Newcastle-Ottawa Scale (NOS) for Assessing the Quality if Nonrandomized Studies in Meta-Analyses. http://www.ohri.ca/programs/clinical_epidemiology/oxford.htm.

[102] Moola S., Munn Z., Tufanaru C., Aromataris E., Sears K., Sfetcu R., Currie M., Qureshi R., Mattis P., Lisy K., Aromataris E., Munn Z. (2017). Chapter 7: Systematic Reviews of Etiology and Risk. Joanna Briggs Institute Reviewer’s Manual.

[103] Aromataris E., Fernandez R., Godfrey C., Holly C., Kahlil H., Tungpunkom P. (2015). Summarizing systematic reviews: Methodological development, conduct and reporting of an Umbrella review approach. Int. J. Evid.-Based Healthc..

[104] Christian C.W., Levin A.V., Council on Child Abuse and Neglect, Section on Ophthalmology, American Association of Certified Orthoptists, American Association for Pediatric Ophthalmology and Strabismus, American Academy of Ophthalmology (2018). The Eye Examination in the Evaluation of Child Abuse. Pediatrics.

[105] Saleh M., Schoenlaub S., Desprez P., Bourcier T., Gaucher D., Astruc D., Speeg-Schatz C. (2009). Use of digital camera imaging of eye fundus for telemedicine in children suspected of abusive head injury. Br. J. Ophthalmol..

[106] Gunda D., Cornwell B.O., Dahmoush H.M., Jazbeh S., Alleman A.M. (2018). Pediatric Central Nervous System Imaging of Non-accidental Trauma: Beyond Subdural Hematomas. RadioGraphics.

[107] Lambert S.R., Johnson T.E., Hoyt C.S. (1986). Optic Nerve Sheath and Retinal Hemorrhages Associated With the Shaken Baby Syndrome. Arch. Ophthalmol..

[108] Luigi Crudele G.D., Galante N., Fociani P., Del Gobbo A., Tambuzzi S., Gentile G., Zoja R. (2021). The forensic application of the Glycophorin A on the Amussat’s sign with a brief review of the literature. J. Forensic Leg. Med..

[109] Geddes J.F. (1997). What’s new in the diagnosis of head injury?. J. Clin. Pathol..

[110] The Ophthalmology Child Abuse Working Party (1999). Child abuse and the eye. Eye.

[111] Delteil C., Humez S., Boucekine M., Jouvet A., Hedouin V., Fanton L., Leonetti G., Tuchtan L., Piercecchi M.-D. (2018). Histological dating of subdural hematoma in infants. Int. J. Leg. Med..

[112] Turillazzi E., Karch S.B., Neri M., Pomara C., Riezzo I., Fineschi V. (2007). Confocal laser scanning microscopy. Using new technology to answer old questions in forensic investigations. Int. J. Leg. Med..

[113] Reichard R.R., White C.L., Hogan R.N., Hladik C.L., Dolinak D. (2004). Beta-amyloid precursor protein immunohistochemistry in the evaluation of pediatric traumatic optic nerve injury. Ophthalmology.

